# Predator Mimicry: Metalmark Moths Mimic Their Jumping Spider Predators

**DOI:** 10.1371/journal.pone.0000045

**Published:** 2006-12-20

**Authors:** Jadranka Rota, David L. Wagner

**Affiliations:** Department of Ecology and Evolutionary Biology, University of Connecticut Storrs, Connecticut, United States of America; University of Exeter, Cornwall Campus, United Kingdom

## Abstract

Cases of mimicry provide many of the nature's most convincing examples of natural selection. Here we report evidence for a case of predator mimicry in which metalmark moths in the genus *Brenthia* mimic jumping spiders, one of their predators. In controlled trials, *Brenthia* had higher survival rates than other similarly sized moths in the presence of jumping spiders and jumping spiders responded to *Brenthia* with territorial displays, indicating that *Brenthia* were sometimes mistaken for jumping spiders, and not recognized as prey. Our experimental results and a review of wing patterns of other insects indicate that jumping spider mimicry is more widespread than heretofore appreciated, and that jumping spiders are probably an important selective pressure shaping the evolution of diurnal insects that perch on vegetation.

## Introduction

**Figure 1:**
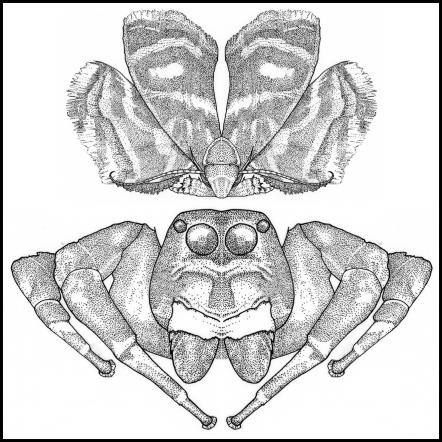
*Brenthia* moths are jumping spider mimics. The moth (upper image) mimics jumping spiders (lower image) with wing markings, wing positioning, posture, and movement (drawing by Virginia Wagner). These moths survive encounters with jumping spiders more often than controls. Moreover, jumping spiders respond to them with territorial displays that are normally directed towards other jumping spiders, indicating that *Brenthia* moths are being mistakenly recognized as jumping spiders, and not as potential prey.

The phenomenon of mimicry, a high degree of resemblance due to selection, was first proposed in 1862 by Sir Walter Henry Bates upon his return from eleven years as a professional collector in Amazon. Writing about butterfly wing patterns, Bates noted, “… on these expanded membranes Nature writes, as on a tablet, the story of the modifications of species…” Bates proposed that longwings and other butterflies gain protection by mimicking distasteful species and that the resemblances among such unrelated insects lent support to Charles Darwin's newly proposed theory of natural selection [1]. Since Bates's initial contribution various cases of mimicry have been identified from across the tree of life. In this paper, we describe a curious form of Batesian mimicry – again involving the wing patterns of Lepidoptera – in which prey (metalmark moths) obtain protection by mimicking their predators (jumping spiders) (Fig. 1).

Many examples of Batesian and Müllerian mimicry and camouflage have been described [1]–[4]. Even cases of aggressive mimicry, where predators mimic prey, are known (e.g., females of *Photuris* fireflies lure males of different firefly species to their death by mimicking their courtship signals [5]). However, predator mimicry – cases in which prey have evolved to mimic their predators to thwart predation attempts – are both exceptional and rare.

Predator mimicry was suggested for owls, where owl ear tufts mimic mammalian predators for protection from such predators as lynx, fox, and marten [6]. Another potential case of predator mimicry is among South American cichlids: coloration and spotting of certain prey species makes them so similar to their predators that they are thought to be their mimics [7]. Eyespots on the wings of giant silk moths and other Lepidoptera undoubtedly mimic eyes of mammalian predators – but here the eyes may function not to mimic their would-be predators, but to resemble a much larger animal, one sizable enough to be a threat to lepidopteran would-be predators. Hence, these eyespots might be regarded as startle coloration [8]. However, in none of these cases are there experimental data demonstrating the efficacy of this mimicry. Our literature review suggests there are few well-supported cases of predator mimicry: e.g., lycaenid butterflies that chemically mimic ants [9] and salticid spiders that mimic ants to avoid being preyed upon by them [10] (other ant mimics probably gain protection from all predators that tend to avoid ants [11]). Here we present evidence for another case of predator mimicry involving salticid spiders, but in this case salticids are predators and not prey.

**Figure 2:**
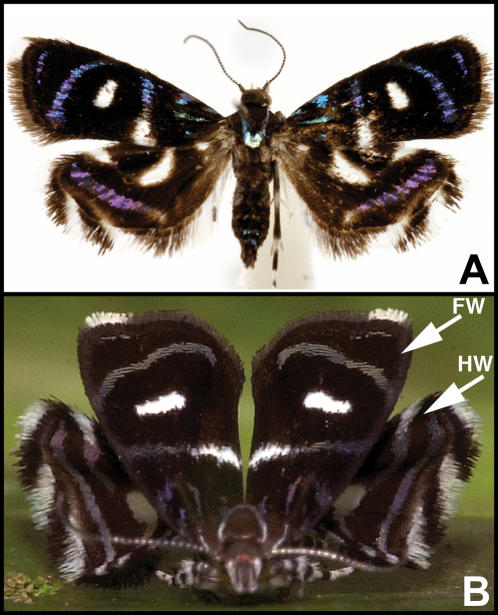
*Brenthia hexaselena*. a, Prepared specimen of *B. hexaselena*. b, Live *B. hexaselena*, FW – forewing, HW – hindwing.

Jumping spiders (Araneae: Salticidae) are visual predators of small arthropods. Among the cues that salticids use for distinguishing between prey, mates, rivals, and enemies are shape, symmetry, presence of legs and wings, size, and style of motion [12]. Their vision is highly acute – they can discriminate between objects even at 40 body lengths [13]. They maintain territories through ritualized displays, which are aimed at both con- and heterospecific individuals [14]. In many salticids, this display consists of a male raising and waving his forelegs at an intruding spider (see video S1 showing two males displaying). If salticids mistakenly recognize potential prey organisms as other jumping spiders, mimetic prey would enjoy higher chances of survival because, instead of an attack, a territorial display may ensue, making it more likely for the prey to escape predation. The results of our study provide evidence that this scenario occurs within *Brenthia* metalmark moths (Fig. 2a and 2b).

## Results/Discussion

All species of *Brenthia* which we observed possess the same adult behavior: during the day both males and females perch on upper surfaces of vegetation and adopt a peculiar posture (Fig. 2b). Their hindwings are fanned outwards and brought forward, perpendicular to the forewings; the forewings are raised and held above the body at approximately a 45° angle [15], [16]. In this position, the alternating white and black fascia on the hindwings are reminiscent of salticid legs (Fig. 1). Moreover, both sexes move with short, rapid, jerky motions, in much the same fashion as jumping spiders (see video S2 of live *B. hexaselena* and *B. monolychna*). Their exceptional wing posture, in conjunction with spiderlike wing markings and movement, makes *Brenthia* moths salticid look-alikes.

**Figure 3:**
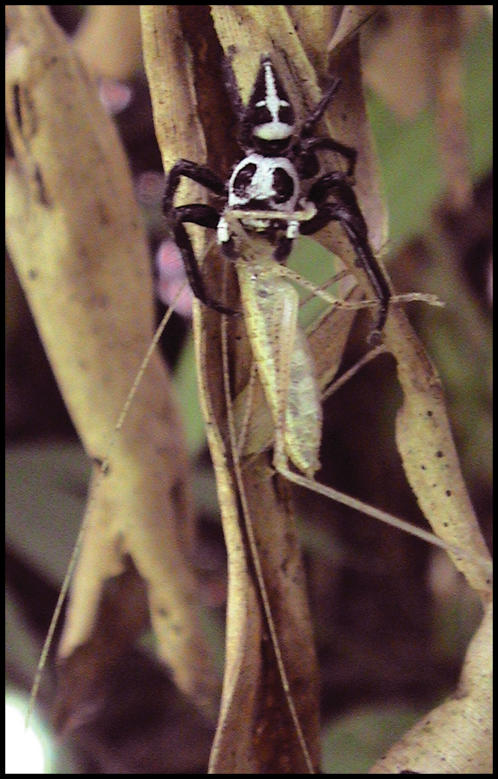
*Phiale formosa*.

In 2003, we tested the hypothesis that *Brenthia* moths are salticid mimics by setting up trials in which one jumping spider (*Phiale formosa*) (Fig. 3) – the largest most common and conspicuous salticid at the study site – was paired with one moth in a small arena. Tested moths were either a presumed mimic (*Brenthia*) or a control species (other choreutids or comparably-sized moths, none of which exhibited wing patterns resembling salticids or engaged in the jerky movements characteristic of *Brenthia*). If *Brenthia* elicited a territorial display from spiders, we concluded that the moths were mistaken for salticids.

We carried out 78 trials (n_mimics_ = 39, n_controls_ = 39). Presumed mimics had much higher rates of survival than controls (*p*<0.0001, G = 18.96) (Table 1), demonstrating an adaptive value to the *Brenthia* morphology and/or behavior. However, the frequency of spider “stalk” and “pounce” behaviors indicated that the mimics were recognized as potential prey as often as controls (*p* = 0.1096, G = 2.56) (Table 1). Not one *P. formosa* made a territorial display, suggesting all spiders used in the trials perceived *Brenthia* moths as prey. The position of wings in *Brenthia* adults may have afforded some protection – when pouncing on a moth, the spiders first hit the raised wings, and, as a consequence, missed the body of the moth. Their resemblance to jumping spiders may have been unimportant.

**Table 1:**
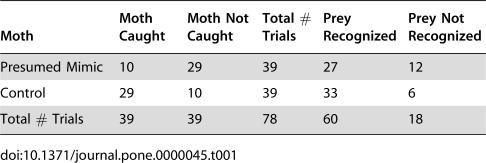
Results of trials with the jumping spider *Phiale formosa* and *Brenthia* (presumed mimic) and control moths.

Moth	Moth Caught	Moth Not Caught	Total # Trials	Prey Recognized	Prey Not Recognized
Presumed Mimic	10	29	39	27	12
Control	29	10	39	33	6
Total # Trials	39	39	78	60	18

Since size has been shown to be a good predictor of the outcome for encounters between jumping spiders [17], one possible explanation for the lack of support for the hypothesis of salticid mimicry in the trials with *P. formosa* is the size difference between the prey and mimic – mature *P. formosa* are four times larger than the *Brenthia* moths used in the trials. To test how aggressive *P. formosa* is towards smaller spiders, we presented it with salticid spiders comparable in size to *Brenthia* moths in four trials. Although the smaller salticids engaged in a territorial display, in all four cases *P. formosa* individuals attacked and killed the smaller spiders without displaying back. These trials suggested that even if *Brenthia* were perfect salticid mimics, *P. formosa* would perceive them as prey.

Based on these results, we returned to the study site in 2005 to repeat our experiments using jumping spiders of similar size to *Brenthia* moths. As in our previous study, *Brenthia* had much higher survival rates when compared to controls: out of 77 *Brenthia* moths, only 5 were caught, whereas 43 of 69 controls were killed (n_trials_ = 146, n_mimic_ = 77, n_control_ = 69, *p*<10^−8^, G = 55.8) (Table 2). More interestingly, in this set of trials many jumping spiders engaged in territorial displays after encountering a *Brenthia*; no spiders displayed to control moths (*p*<10^−7^, G = 33.96) (Table 2). In no trials in which spiders displayed toward *Brenthia* did *Brenthia* get caught. The difference in response to *Brenthia* versus controls was marked: spiders began stalking control moths soon after an individual was introduced into the arena and, usually after only a few pounces and without hesitation, caught and fed on the non-mimetic prey (see video S3 showing five trials with control moths). In trials with *Brenthia* moths the spiders first observed the moth, and then many (in 36% of the trials) raised and waved their forelegs towards the moth, i.e., engaged in territorial behavior (see video S4 of five trials with *Brenthia* moths). In several trials (n_trials_ = 11), spiders even backed away from the mimics, demonstrating that many *Brenthia* are perceived as salticids – their ploy of donning wolf's clothing proves successful.

**Table 2:**
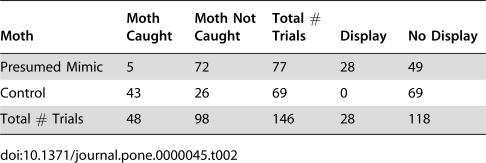
Results of trials with 12 species of smaller jumping spiders and *Brenthia* (presumed mimic) and control moths.

Moth	Moth Caught	Moth Not Caught	Total # Trials	Display	No Display
Presumed Mimic	5	72	77	28	49
Control	43	26	69	0	69
Total # Trials	48	98	146	28	118

The adaptive value of jumping spider mimicry may extend beyond escaping predation by salticids. Mimicking jumping spiders may be helpful against birds and other vertebrate predators through evasive prey mimicry, wherein protection is gained through resemblance to prey that is too costly for predators to capture [18]–[20]. For example, if birds learn that it is not worthwhile to pursue salticids as prey, because these wary spiders often evade capture, then credible salticid mimics would be ignored as well. Lindroth [18] proposed this form of mimicry as the explanation for why some ground beetles in the genus *Lebia* (Coleoptera: Carabidae) resemble flea-beetles (Coleoptera: Chrysomelidae: Alticinae) and Balgooyen [19] suggested it as an explanation for why some short-horned grasshoppers mimic the alfalfa butterfly (*Colias eurytheme*). While no empirical studies have been conducted to support this mimicry type, Ruxton et al. [20] demonstrated mathematically that evasive prey mimicry would be selectively advantageous when there is alternative prey available and the evasiveness of the model is costly to the predator. We consider it likely that *Brenthia* and other insects that are salticid spider mimics are also favored through evasive prey mimicry, as well as predator mimicry.

Jumping spider mimicry appears to be taxonomically widespread among insects. Tephritid flies that mimic salticids have higher survival rates in encounters with jumping spiders when compared to other similar-sized, non-mimetic flies [21]–[23]. Salticid mimicry has been suggested for three fulgoroid homopterans: an issid [24], a fulgorid [25], and a derbid [26]. Both wing patterns and perching behaviors of other lepidopterans, including nymphuline pyralids, hilarographine tortricids, glyphipterigids, *Fabiola* (in the Oecophorinae), *Ecballagonia bimetallica* (in the Cosmopterigidae), and scattered gelechiid genera are suggestive of jumping spider mimicry. In addition to having lightly-coloured fascia along the leading edge of the forewing (which yield the impression of legs), these microlepidopteran taxa possess groups of eyespots, frequently including metallic scales, along the trailing edge of the forewing or an exposed, often elevated, portion of the hindwing that collectively resemble the clustered eyes of a salticid spider. As in *Brenthia*, the postures of the mimics are often dramatically showy relative to those of their non-mimetic sister taxa. The convergent acquisition of grouped eyespots and leg-like markings on wings of small moths and other insects offers further evidence that the vision of some invertebrates is significantly more sophisticated than generally appreciated. Presumably, the highly acute vision of jumping spiders is an integral part of the selective force shaping these mimicry systems. If even a portion of the various instances of salticid mimicry in the Diptera, Homoptera, and those proposed here for the Microlepidoptera prove to be valid, then jumping spiders can be regarded as important drivers in the evolution of diurnal insect phenotypes and behaviors.

## Materials and Methods

This project was carried out at La Selva Biological Station (Province of Heredia, Costa Rica) in March and July of 2003 and August and September of 2005. Four species of *Brenthia* metalmark moths were used as mimics: *B. hexaselena* Meyrick, *B. monolychna* Meyrick, and two undescribed species. Voucher specimens of the four *Brenthia* are deposited in the University of Connecticut Entomological Collection (UCMS). *Brenthia* were collected during the day from vegetation.

Individuals from several lepidopteran families were used as controls. A moth qualified for control trials if it was similar in size to *Brenthia* and did not resemble jumping spiders. Control moths were obtained from mercury vapor light and diurnal net collections or by rearing. All moths were used in a single trial within 24 hours of collection or emergence.

In trials that were done in 2003, we used 78 individuals of the jumping spider *Phiale formosa* (Banks). In 2005 trials we used 80 individuals representing 12 species of jumping spiders that were similar in size to *Brenthia* (body length 5–8 mm). Vouchers of all spider individuals used in 2005 trials are deposited in the UCMS collection. Spiders were held in 15-dram plastic vials and fed small arthropods initially, then kept without food for 48 h before trials. In 2003 each spider was used only once. In 2005, 62 of 80 spiders were used in two trials, once with a mimic and once with a control; 12 were used with mimics only; 4 were used with controls only; and 2 were used in 3 trials (either with 2 mimics and 1 control or *vice versa*). When used in more than one trial, spiders were randomly assigned first to mimic or control trials and they were held for at least two days between successive trials. Both male and female moths and spiders were used in the trials.

Trials in 2003 were carried out in a plastic arena (13×8×7 cm) with a transparent top; in 2005 we used a slightly smaller Plexiglas® arena (10×5×4 cm). In every trial, a spider was first placed into the arena and then later the moth was introduced. Trials were terminated upon capture of the moth or after 5 minutes. Four behaviors were recorded: 1. “stalk” – spider stealthily approached the moth; 2. “pounce” – spider jumped towards the moth and attempted to catch it; 3. “catch” – spider captured the moth; 4. “display” – spider raised and waved its forelegs. All trials in 2005 were filmed with a mini DV camera and reviewed afterwards. Video footage of five trials with mimics and five trials with controls is available as supporting information, as well as footage of live *B. hexaselena* and *monolychna* and spider territorial displays. All video footage was made at La Selva.

In 2003 we carried out 78 trials, 39 with mimics and 39 with controls, and in 2005, 146 trials, 77 with mimics and 69 with controls. The data were organized into a 2×2 contingency table with the marginal totals for the number of trials fixed (Tables 1 and 2). We analyzed the data with the G-test of independence, applying Williams's correction [27]. For the calculation of the G value for data shown in Table 2, we replaced the 0 value for the number of displays to control moths with the number 1 to facilitate calculation.

## Supporting Information

Video S1 Territorial behavior of two male salticids.
(13.55 MB MOV)

Video S2 Display of live *B. hexaselena* and *monolychna*.
(2.00 MB MOV)

Video S3 Five trials with salticids and control moths.
(11.23 MB MOV)

Video S4 Five trials with salticids and *Brenthia* moths.
(17.17 MB MOV)

## References

[1] Bates HW (1862). Contributions to an insect fauna of the Amazon Valley. Lepidoptera: Heliconidae.. Trans Linn Soc Lond.

[2] Müller F (1879). Ituna and Thyridia: a remarkable case of mimicry in butterflies.. Proc Entomol Soc.

[3] Rettenmeyer CW (1970). Insect mimicry.. Annu Rev Entomol.

[4] Owen D (1980). Camouflage and Mimicry.

[5] Lloyd JE (1965). Aggressive mimicry in *Photuris*: firefly femmes fatales.. Science.

[6] Perrone M (1981). Adaptive significance of ear tufts in owls.. Condor.

[7] Zaret TM (1977). Inhibition of cannibalism in *Cichla ocellaris* and hypothesis of predator mimicry among South American fishes.. Evolution.

[8] Stevens M (2005). The role of eyespots as anti-predator mechanism, principally demonstrated in the Lepidoptera.. Biol Rev.

[9] Akino T, Knapp JJ, Thomas JA, Elmes GW (1999). Chemical mimicry and host specificity in the butterfly *Maculinea rebeli*, a social parasite of *Myrmica* ant colonies.. Proc R Soc Lond B.

[10] Nelson XJ, Jackson RR, Edwards GB, Barrion AT (2005). Living with the enemy: jumping spiders that mimic weaver ants.. J Arachnol.

[11] McIver JD, Stonedahl G (1993). Myrmecomorphy: morphological and behavioral mimicry of ants.. Annu Rev Entomol.

[12] Jackson RR, Pollard SD (1996). Predatory behavior of jumping spiders.. Annu Rev Entomol.

[13] Harland DP, Jackson RR, Macnab AM (1999). Distances at which jumping spiders (Araneae: Salticidae) distinguish between prey and conspecific rivals.. J Zool.

[14] Jackson RR, Witt PN, Rovner JS (1982). The behavior of communicating in jumping spiders (Salticidae).. Spider Communication Mechanisms and Ecological Significance..

[15] Clemens B (1860). Contributions to American lepidopterology. No. 4.. Proc Acad Nat Sci Philadelphia.

[16] Aiello A, Becker VO (2004). Display of the “Peacock Moth”: *Brenthia* spp. (Choreutidae: Brenthiinae).. J Lep Soc.

[17] Wells MS (1998). Effect of body size and resource value on fighting behaviour in a jumping spider.. Anim Behav.

[18] Lindroth CH (1971). Disappearance as a protective factor: a supposed case of Bates'ian [sic] mimicry among beetles (Coleoptera: Carabidae and Chrysomelidae).. Ent Scand.

[19] Balgooyen TG (1997). Evasive mimicry involving a butterfly model and grasshopper mimic.. Am Mid Nat.

[20] Ruxton GD, Speed M, Sherratt TN (2004). Evasive mimicry: when (if ever) could mimicry based on difficulty of capture evolve?. Proc R Soc Lond.

[21] Greene E, Orsak LJ, Withman DW (1987). A tephritid fly mimics the territorial display of its jumping spider predators.. Science.

[22] Mather MH, Roitberg DB (1987). A sheep in wolf's clothing: tephritid flies mimic spider predators.. Science.

[23] Whitman DW, Orsak L, Greene E (1988). Spider mimicry in fruit flies (Diptera: Tephritidae): further experiments on the deterrence of jumping spiders (Araneae: Salticidae) by *Zonosemata vittigera* (Coquillett).. Ann Entomol Soc America.

[24] O'Brien LB (1967). *Caliscelis bonellii* (Latreille), a European genus of Issidae new to the United States (Homoptera: Fulgoroidea).. Pan-Pac Entomol.

[25] Zolnerowich GA (1992). Unique *Amycle* nymph (Homoptera: Fulgoridae) that mimics jumping spiders (Araneae: Salticidae).. J New York Entomol Soc.

[26] Floren A, Otto S (2001). A tropical Derbidae (Fulgoroidea, Homoptera) that mimics a predator (Salticidae, Araneae).. Ecotropica.

[27] Sokal RR, Rohlf FJ (1981). Biometry: The Principles and Practice of Statistics in Biological Research,.

